# Response of Bacterial Community to the Occurrence of Clubroot Disease in Chinese Cabbage

**DOI:** 10.3389/fmicb.2022.922660

**Published:** 2022-07-06

**Authors:** Haiping Ni, Rui Zong, Jianjun Sun, Yuxia Wu, Lei Yu, Yuanyuan Liu, Jin Liu, Ruicheng Ju, Xianli Sun, Yulian Zheng, Lekun Tan, Lumin Liu, Yachao Dong, Tao Li, Youming Zhang, Qiang Tu

**Affiliations:** ^1^Helmholtz International Lab for Anti-Infectives, State Key Laboratory of Microbial Technology, Shandong University–Helmholtz Institute of Biotechnology, Shandong University, Qingdao, China; ^2^Qingdao Hexie Biotechnology Co., Ltd., Qingdao, China; ^3^Shandong Agricultural Technology Extension Center, Jinan, China; ^4^Chinese Academy of Sciences (CAS) Key Laboratory of Quantitative Engineering Biology, Shenzhen Institute of Synthetic Biology, Shenzhen Institute of Advanced Technology, Chinese Academy of Sciences, Shenzhen, China

**Keywords:** Chinese cabbage, clubroot disease, bacterial community, diversity, structure, differential bacteria

## Abstract

Clubroot disease is a common soilborne disease caused by *Plasmodiophora brassicas* Wor. and widely occurs in Chinese cabbage. Soil microorganisms play vital roles in the occurrence and development of plant diseases. The changes in the soil bacterial community could indicate the severity of plant disease and provide the basis for its control. This study focused on the bacterial community of the clubroot disease-infected soil–root system with different severity aiming to reveal the composition and structure of soil bacteria and identified potential biomarker bacteria of the clubroot disease. In the clubroot disease-infected soil, the bacterial community is mainly composed of *Actinobacteria, Gammaproteobacteria, Alphaproteobacteria, Bacilli, Thermolrophilia, Bacteroidia, Gemmatimonadetes, Subgroup_6*, Deltaproteobacteria, *KD4-96*, and some other classes, while the major bacterial classes in the infected roots were *Oxyphotobacteria, Gammaproteobacteria, Alphaproteobacteria, Actinobacteria, Bacilli, Bacteroidia, Saccharimonadia, Thermoleophilia, Clostridia, Chloroflexia*, and some other classes. The severe clubroot disease soil–root system was found to possess a poorer bacterial richness, evenness, and better coverage. Additionally, a significant difference was observed in the structure of the bacterial community between the high-severity (HR) and healthy (LR) soil–root system. *Bacillus asahii* and *Noccaea caerulescens* were identified as the differential bacteria between the LR and HR soil and roots, respectively. pH was demonstrated as a vital factor that was significantly associated with the abundance of *B. asahii* and *N. caerulescens*. This study provides novel insight into the relationship between soil bacteria and the pathogen of clubroot disease in Chinese cabbage. The identification of resistant species provides candidates for the monitoring and biocontrol of the clubroot disease.

## Introduction

Chinese cabbage is one of the most essential vegetables in the daily diet of Chinese people, which has a planting history of over 3,000 years. Clubroot disease is a soilborne disease induced by the *Plasmodiophora brassicas* Wor., which widely affects the brassicaceous vegetables (Si et al., 2002; Li et al., 2013; Strehlow et al., 2015). With the alternation of vegetable breeds and infection of various fungi and bacteria, the clubroot disease was primarily discovered 280 years ago (Heo et al., 2009). Chinese cabbage is highly susceptible and greatly influenced by the clubroot disease. According to previous data, the incidence of clubroot disease in Chinese cabbage has increased to over 60% (Zhang et al., 2019; Hasan et al., 2021). The common symptoms of clubroot-infected vegetables were swollen roots, wilting overground parts, and stationary growth, which seriously limited the yield and quality of the vegetables (Hwang et al., 2012). Due to the long survival of dormant sporangium in the soil, there is a lack of effective methods to totally eradicate clubroot disease. Therefore, the measures of the precaution and control of *P. brassicas* have attracted special attention.

The control of clubroot has become a challenge for a long period and has attracted global attraction. Currently, the general steps for the prevention of clubroot disease include agricultural chemicals, liming, and biocontrol (Ahmed et al., 2020; Hasan et al., 2021; Xi et al., 2021). Chemicals have been considered the most effective control means, but they were demonstrated to possess the limitation of resistance, pollution, and decreased food quality. Numerous research has concentrated on the biocontrol of clubroot disease, which is more sustainable and environmentally friendly, and a number of biocontrol strains of clubroot disease have been dug out. For example, a previous study found four *lysobacter* strains that showed significant inhibitory effects on the clubroot disease to varying degrees (Fu et al., 2018). Another field study demonstrated the dramatically inhibitory effect of the *Streptomyces alfalfa* XY25^*T*^ on the clubroot disease by changing the rhizosphere microbial community (Hu et al., 2021). The inoculation of *Trichiderma harzianum* was suggested to regulate the rhizosphere microbial community and therefore suppressed the incidence of clubroot disease in Chinese cabbage (Li J. et al., 2020). However, most studies focused on the effect of exogenous and overlooked the critical role of soil-isolated microorganisms in the control of clubroot disease.

The composition of soil microorganisms is closely associated with the nutrient cycle and the structure of the soil, which could further affect the emergence and propagation of plant disease (Santhanam et al., 2015; Daval et al., 2020). The higher microbial diversity could provide stronger protection to plants from the invasion of adverse diseases. Recently, increasing studies have been devoted to disclosing the effect of plant disease on soil microorganism compositions, aiming to reveal the association between microbial community and the onset of plant disease and identify disease-related microbiomes (Cheng et al., 2019; Shi et al., 2019; Ding et al., 2021). The biocontrol of soilborne disease was based on the identification of key microbiomes related to plant diseases and the development of engineering bacteria to control the pathogenesis and progression of those diseases.

With the development of bioinformatics, the changes in the microbial community of plant disease-infected soil have become an effective channel to evaluate the development and potential of plant disease (Wu et al., 2014; Balbín-Suárez et al., 2021; Chen et al., 2022). This study focused on the microbial composition of clubroot disease-infected soil. By comparing the difference in soil microbial community between the high-severity (HS) soil and healthy soil, the characteristics of clubroot-infected soil were disclosed. Additionally, with the help of high-throughput sequencing, the composition and structure of soil microbiomes associated with clubroot disease were declaimed, which could predict the occurrence of clubroot and develop potential engineering bacteria for its biocontrol.

## Materials and Methods

### Field Experiment and Sampling

The field experiment was conducted at Jiaoheyuan Cabbage Base, Puji Town, Jiaozhou City, Shandong Province (36.11°N, 119.71°E). The selected field includes an infected area and a healthy area. According to the disease index of the cabbages, the field was grouped as HR (disease index ≥ 85%) and healthy group (LR, disease index < 10%). The Chinese cabbage was transplanted at the five-leaf stage on 8 September 2020, in rows 50 cm apart, and the distance between the plants was 40 cm.

The plants were sampled on 4 November 2020, 56 days after transplanting. Five plants were collected from each group and stored in the ice bag. The collected plants had to be sent to the laboratory in 12 h. The roots were washed with the sterile PBS buffer and centrifuged to obtain the root-attached soil. The collected soil samples were stored at −80°C for the following analyses, and some fresh samples were used in the physicochemical analyses.

### Physiochemical Analysis

The physicochemical features of the collected soil samples, including soil organic matter (SOM), total nitrogen (TN), moisture content, ammonium (${\text{NH}}_{4}^{+}$N), nitrate (${\text{NO}}_{3}^{-}$N), available phosphorus (AP), available potassium (AK), and electrical conductance (EC) were analyzed according to previous reports at the ratio of 1:2.5 (soil:CO_2_-free water, w/v).

### DNA Isolation and PCR

DNA in the soil samples was isolated with the E.N.Z.A.™ Soil DNA Kit (Omega, USA) according to the protocols of the manufacturer. The quality and quantity of isolated DNA were evaluated with the NanoDrop 2000 spectrophotometer (Thermo Scientific, USA).

The primers 515F (5′ -GGACTACVSGGGTATCTAAT-3′ ) and 806R (5′ -GTGCCAGCMGCCGCGGTAA-3′ ) were used for the amplification of the bacterial V3–V4 regions with the reaction conditions as follows: 95°C for 30 min, followed by 30 cycles at 95°C for 30 s; then, annealing at 55°C for 30 s and elongation at 72°C for 30 s; finally, extension at 72°C for 10 min. The reaction volume was 20 μl, which contained 5 μl DNA, 0.2 μl primers (10 μM), 10 μl SYBR reagent, and 4.8 μl ultra-pure water in order to maintain the volume. The quantity of the obtained products was conducted using the QuantiFluor™-ST (Promega, USA) based on the instrument's procedure.

### Illumina MiSeq Sequencing and Data Preceding

The Illumina sequencing was performed with a MiSeq PE250 sequencer (Illumina, USA). The VSEARCH method (Edgar 2011) was applied for the stitching, filtering, deduplication, and clustering of the data. With the help of the UPARSE algorithm, the sequences with similarity of over 97% were classified into the same operational taxonomic unit (OTU). Then, the sequences were classified and annotated to establish the taxonomic tree and phylogenetic tree. The alpha- and beta-diversity indexes were calculated with the rarefied OTUs. The alpha-diversity index includes Chao1, species, Shannon, Simpson, Faith, Pielou, and coverage, which were calculated with the R-package in QIIME2. For further beta-diversity analysis, the principal coordinate analysis (PCoA) was conducted with four distance algorithms. The sequence data were submitted to NCBI Sequence Read Archive (http://www.ncbi.nlm.nih.gov/bioproject/844280) with accession number PRJNA 844280.

### Statistical Analysis

The difference in soil physiochemical features between the LR and HR groups was analyzed with Student's *t*-test using the SPSS 20.0 software. The difference in alpha-diversity between groups was evaluated using Kruskal–Wallis rank and Dunn's tests. The PCoA was conducted based on Python, while the correlation heatmap was obtained using RDA analysis. *p* < 0.05 indicates statistically significant difference.

## Results

### Soil Physicochemical Properties and Gene Sequencing

**Figure 1:**
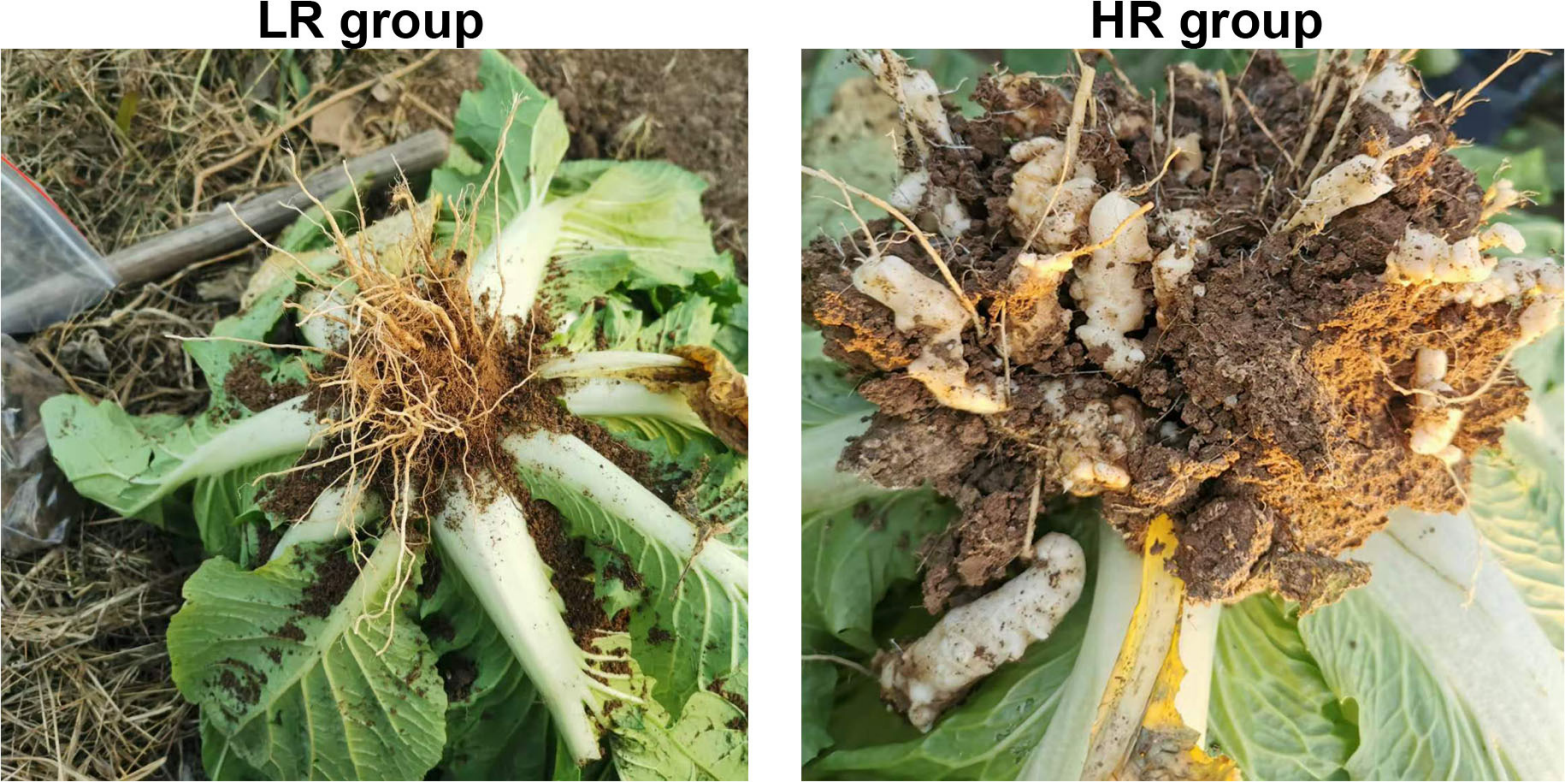
The morphology of the cabbage roots in the LR **(left)** and HR **(right)** groups.

The cabbage roots from the HR group showed obviously swollen roots than the cabbages in the LR group (Figure 1). The basic physicochemical properties of LR and HR soil are summarized in Table 1. A significant increase was observed in the AK and EC of HR soil compared with the LR soil (*p* < 0.05), while other properties, such as SOM, TN, moisture content, ${\text{NH}}_{4}^{+}$N, ${\text{NO}}_{3}^{-}$N, AP, and pH, showed no significant difference between the LR and HR soil (*p* > 0.05).

**Table 1:** Basic physicochemical properties of soil.

	**LR soil**	**HR soil**
SOM (g/kg)	27.85 ± 0.80	31.19 ± 1.82
TN (g/kg)	0.99 ± 0.041	1.31 ±0.11
Moisture content (%)	10.12 ± 0.97	16.74 ± 2.99
${\text{NH}}_{4}^{+}$-N (mg/kg)	5.28 ± 0.52	7.77 ± 0.65
${\text{NO}}_{3}^{-}$-N (mg/kg)	21.51 ± 1.72	28.51 ± 2.40
AP (mg/kg)	109.30 ± 10.14	128.50 ± 9.42
AK (mg/kg)	234.10 ± 35.15	448.40 ± 64.27^***^
pH	6.58 ± 0.16	6.84 ± 0.21
EC (μs/cm)	134.5 ± 67.30	206.1 ±64.19^**^

** *p < 0.01,*

*** *p < 0.001 relative to the LR group*.

A total of 514,042 merged sequences were observed after the paired sequence reads merged with the average sequence of 51,404 per sample, and the length of reads ranged from 135 to 440 bp.

### The Bacterial Composition of Soil and Roots in LR and HR Soil and Cabbage Roots

**Figure 2:**
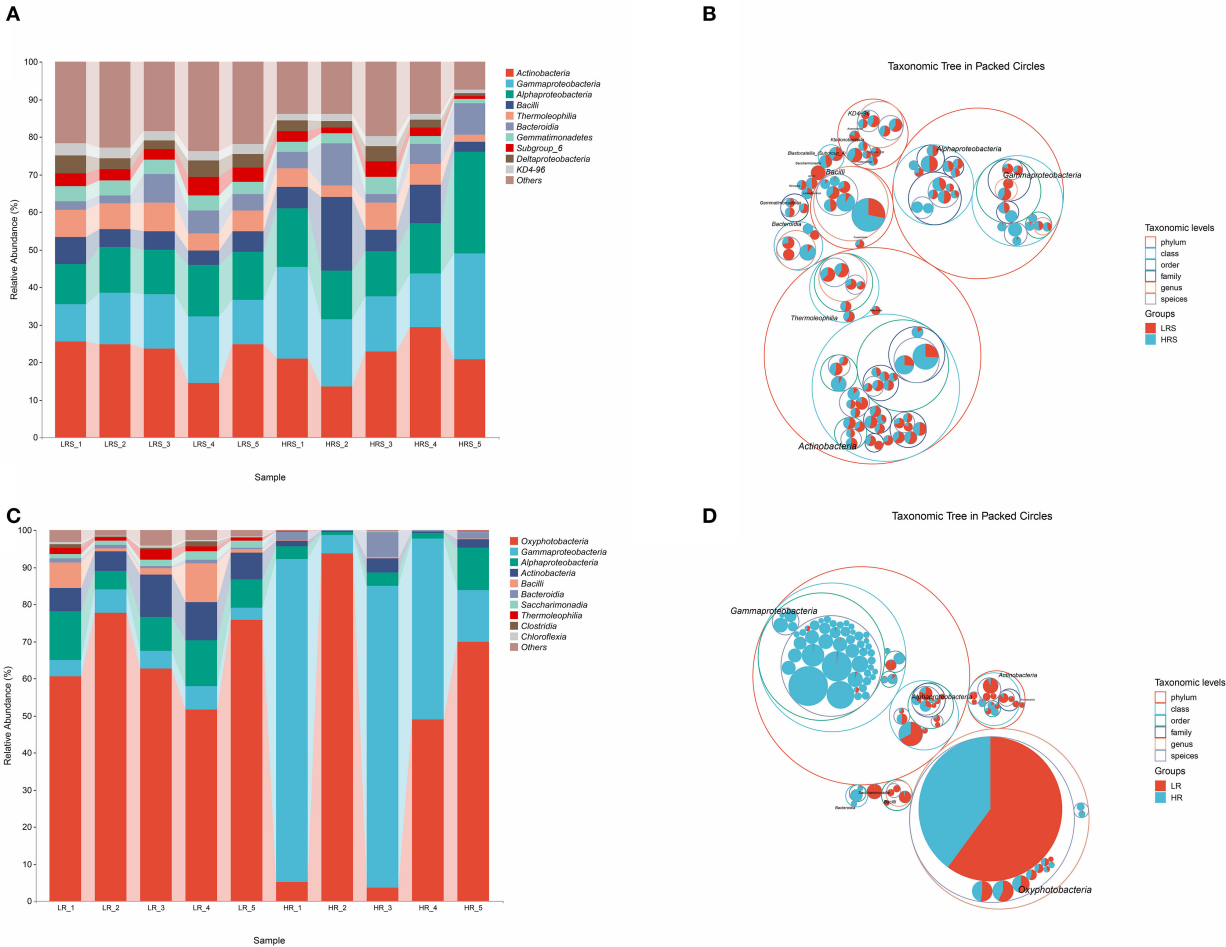
The bacteria community composition in the LR and HR soil–root system. **(A,B)** The relative abundance of classes **(A)** and the taxonomic difference **(B)** between the LR and HR soil. **(C,D)** The relative abundance of classes **(C)** and the taxonomic difference **(D)** between the LR and HR cabbage roots. The largest circle represents the phylum level, and the inter circles represent class, family, and genus.

The bacterial composition of soil and cabbage roots was compared between the LR and HR groups according to the class levels. In the soil of both LR and HR groups, the bacterial composition included *Actinobacteria, Gammaproteobacteria, Alphaproteobacteria, Bacilli, Thermolrophilia, Bacteroidia, Gemmatimonadetes, Subgroup_6*, Deltaproteobacteria, *KD4-96*, and some other classes (Figure 2A), while significant differences were observed in the percentage of *Gammaproteobacteria, Alphaproteobacteria*, and *Bacilli* classes, which possessed a relatively higher proportion in the HR group (*p* < 0.05, Figure 2B).

For the bacterial composition of cabbage roots, *Oxyphotobacteria, Gammaproteobacteria, Alphaproteobacteria, Actinobacteria, Bacilli, Bacteroidia, Saccharimonadia, Thermoleophilia, Clostridia, Chloroflexia*, and some other classes were identified in the cabbage root in the LR group. The bacterial community of the HR cabbage roots only involved *Oxyphotobacteria, Gammaproteobacteria, Alphaproteobacteria, Actinobacteria*, and *Bacteroidia* classes (Figure 2C). Moreover, in the bacterial composition of HR cabbage roots, a relatively higher proportion of *Gammaproteobacteria* and *Bacteroidia* was revealed compared with that of the proportion of LR cabbage roots (*p* < 0.05, Figure 2D). While the proportion of *Oxyphotobacteria* was significantly smaller in the HR cabbage roots, it was lower than that of the LR cabbage roots (*p* < 0.05, Figure 2D). No significant differences were observed in the proportion of the rest classes between the LR and HR soil (*p* > 0.05).

### Response of Alpha-Diversity to LR and HR in Soil and Cabbage Roots

**Figure 3:**
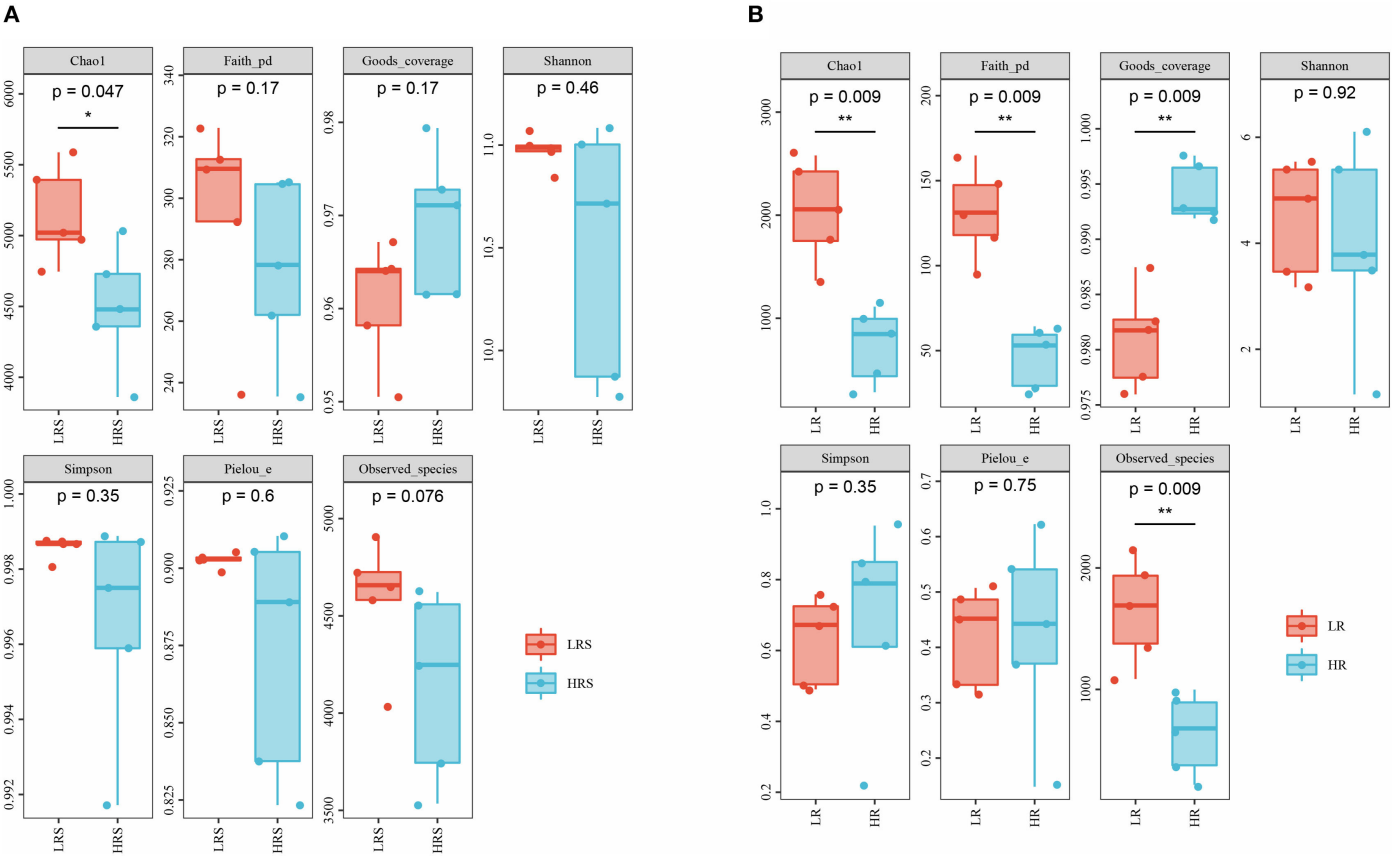
The difference in the alpha-diversity indexes between the LR and HR soil **(A)** and cabbage roots **(B)**. *p < 0.05, **p < 0.01.

The richness, diversity, and evenness of the bacteria in the soil and cabbage root were assessed by alpha-diversity. Compared with the LR soil, the HR soil possessed a significantly lower value of Chao1, indicating the relatively lower bacterial richness in the HR soil (*p* < 0.05, Figure 3A). While an insignificant increase was observed in the coverage index, no significant difference was observed in the Simpson, Pielou, Faith PD, Shannon index, and observed species, which represented the indiscriminate bacterial coverage, diversity, evolution diversity, and evenness between the LR and HR soil (*p* > 0.05, Figure 3A).

In the cabbage roots, the HR roots showed a reduced Chao1 and Faith PD index, while the coverage index was significantly higher than that of LR roots (*p* < 0.05, Figure 3B). The difference in Simpson, Pielou, Shannon index, and Observed species between the LR and HR roots was not significant (*p* > 0.05, Figure 3B) Interestingly, except for the coverage index, all other corresponding indexes of cabbage roots were significantly lower than those of soil, suggesting the relative poorer bacterial richness, diversity, and evenness of cabbage roots relative to the soil, while the coverage index was significantly higher in the cabbage roots, indicating the relatively larger bacterial coverage (*p* < 0.05, Supplementary Figure 1).

### Response of Beta-Diversity to LR and HR in Soil and Cabbage Roots

**Figure 4:**
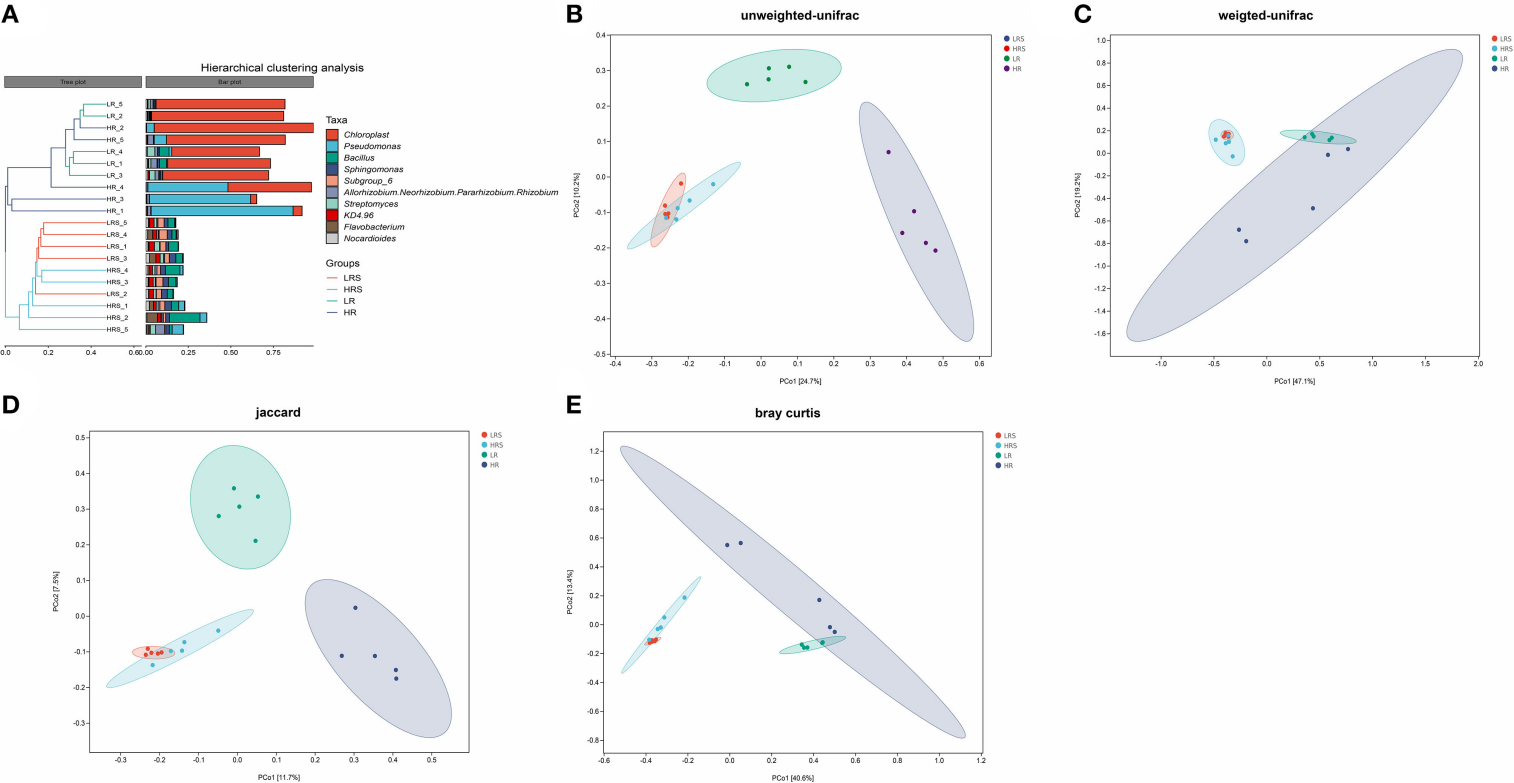
The difference in the bacterial community structure between the LR and HR soil–root system. **(A)** The hierarchical clustering analysis of the LR and HR soil and roots is based on the class level. **(B–E)** Principal coordinate analysis (PCoA) is based on the unweighted UniFrac algorithm **(B)**, weighted UniFrac algorithm **(C)**, Jaccard algorithm **(D)**, and Bray–Curtis algorithm **(E)**.

The beta-diversity indicated the bacterial community structure in the soil and cabbage roots. It was found that the bacterial community structure was completely different between soil and cabbage roots. Specifically, there were two independent clusters observed in the hierarchical clustering tree, which was separated according to the soil and cabbage roots (Figure 4A). Moreover, the results of PCoA based on different algorithms showed distinct results. In the UniFrac results, the unweighted UniFrac algorithm showed a clear separation between the LR roots and HR roots with PC1 and PC2 of 24.7 and 10.2%, respectively (Figure 4B), which was not entirely separated in the weighted UniFrac algorithm (Figure 4C), while the distance between the LR and HR soil was not clear in the unweighted UniFrac and was overlapped in weighted UniFrac (Figures 4B,C). The jaccard algorithm also demonstrated the completed separation of HR roots from the LR roots, where the variation of PC1 was 11.7% and PC2 was 7.5% (Figure 4D). According to the results of Bray–Curtis, the separation between LR and HR soil and roots was not clear with PC1 of 40.6% and PC2 of 13.4% (Figure 4E).

### The Differential Bacteria and Their Correlation With Soil Physicochemical Properties

**Figure 5:**
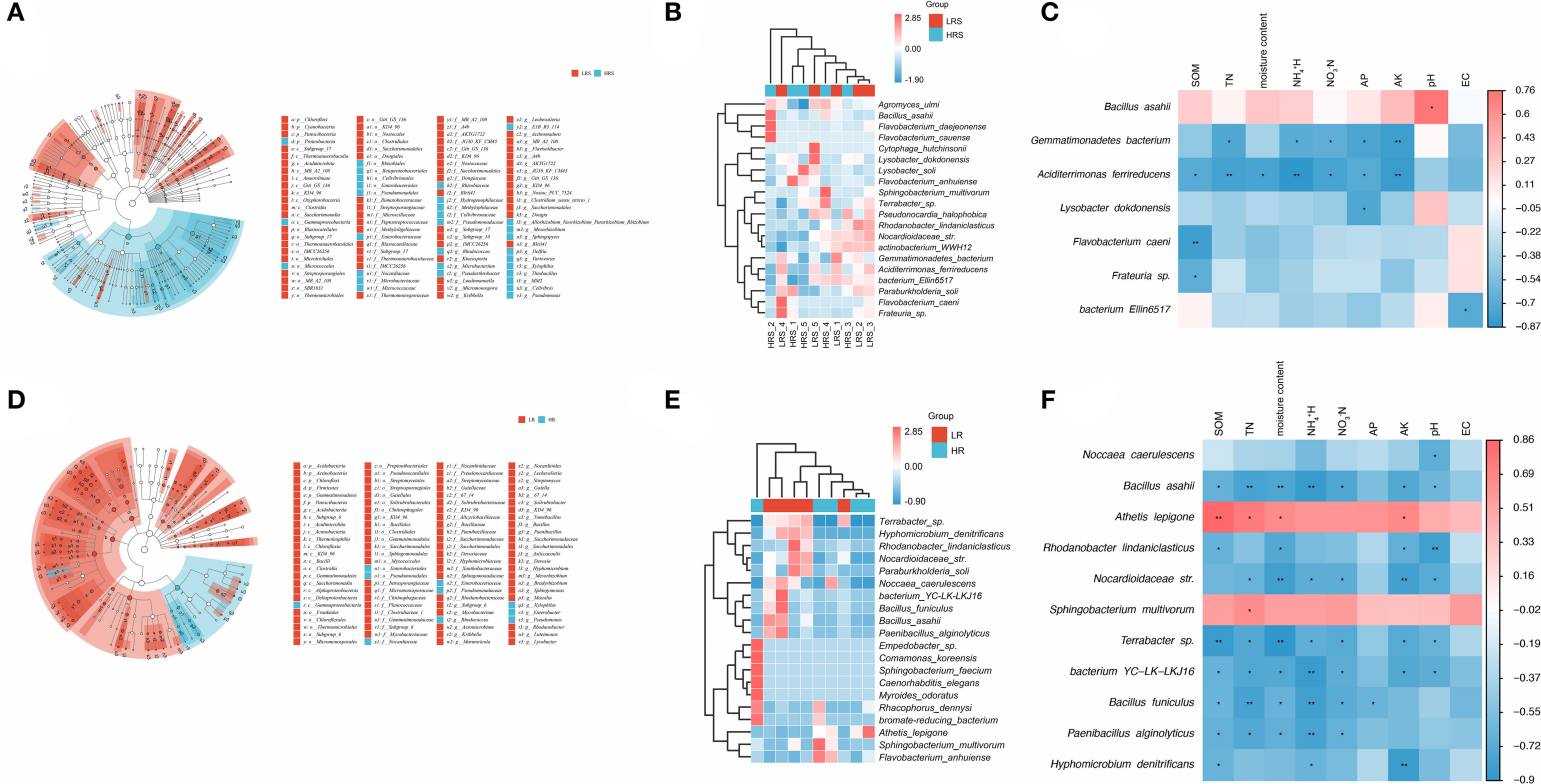
**(A–C)** Significant differential bacterial taxa **(A)** and species **(B)** between the LR and HR soil. The correlation of abundant bacteria in the HR soil with soil physicochemical properties was evaluated with Pearson's correlation analysis **(C)**. **(D,E)** Significant differential taxa **(D)** and genus **(E)** between the LR and HR cabbage roots. The correlation of abundant bacteria in the HR roots with soil physicochemical properties was evaluated with Pearson's correlation analysis **(F)**. Different colors represent the different samples **(A,D)**, abundance **(B,E)**, or correlations **(C,F)**. *p < 0.05, **p < 0.01.

The relative taxonomic abundance of correlated bacteria in the LR and HR soil is shown in Figure 5A. Compared with the LR soil, the mainly enriched taxa in HR soil were found in the *Proteobacteria, Gammaproteobacteria*, and *Micrococcales*. Meanwhile, the differential bacterial genus was identified as *Bacillus* and *Pseudomonas* (Supplementary Figure 2A). The dominant bacterium in the HR soil was identified as the *Bacillus asahii* (Figure 5B). A significant positive correlation between *B. asahii* with soil pH was observed (*p* < 0.05, Figure 5C). Notably, the soil TN, ${\text{NH}}_{4}^{+}$N, ${\text{NO}}_{3}^{-}$N, AP, and AK were found to be negatively correlated with the abundance of *Gemmatimonadetes bacterium*, while the abundance of *Aciditerrimonas ferrireducens* was negatively associated with SOM, TN, moisture content, ${\text{NH}}_{4}^{+}$N, ${\text{NO}}_{3}^{-}$N, AP, and AK (*p* < 0.05, Figure 5C), although there were no significant differences observed in their abundance between the LR and HR soil (*p* > 0.05).

For the bacterial community of cabbage roots, the taxa mainly enriched in the HR roots was found in *Gammaproteobacteria* (Figure 5D). The differential bacterial genus between the LR and HR roots was dug out as *Pseudomonas* and *Chloroplast* (Figure 2B). *Noccaea caerulescens* was found to enrich the HR cabbage roots (Figure 5E), which was negatively correlated with soil pH (*p* < 0.05, Figure 5F). For the other insignificant bacteria, the physiochemical properties also showed varying degrees of promoted or inhibitory effects on their abundance, but soil EC showed no significant correlation with the abundant bacteria in the clubroot disease-infected cabbage roots.

There are some limitations to our study, such as lack of fungi analysis and function enrichment.

## Discussion

As one of the most common soilborne diseases, the infection of clubroot disease in Chinese cabbage has become a challenging problem, which dramatically influenced the yield and quality of plants. Bacterial composition and structure are vital factors closely associated with soil health and further affected plant health. In this study, the bacterial characteristics of clubroot disease-infected soil and cabbage roots were investigated to assess the potential association between bacterial community and clubroot disease severity.

### Response of Bacterial Composition to Clubroot Disease in the Infected Soil and Cabbage Roots

In clubroot disease-infected soil, the bacterial community was similar between the LR and HR soil according to the class level. However, the specific proportion of various classes was significantly different. It was observed that the HR soil possessed a high proportion of *Gammaproteobacteria, Alphaproteobacteria*, and *Bacilli* classes. Similarly in the HR cabbage roots, a large abundance of *Gammaproteobacteria* was also observed, together with *Bacteroidia*. Moreover, the reduced abundance of *Oxyphotobacteria* was noted in the HR cabbage roots. Previously, the advantage of *Gammaproteobacteria, Bacteroidia*, and *Alphaproteobacteria* and the difference in the abundance of *Oxyphotobacteria* in the rhizosphere soil of Crucifer clubroot disease were revealed, which was consistent with the present results (Wu et al., 2020).

The elevating abundance of *Gammaproteobacteria* in both HR soil and cabbage roots suggested the high resistance of *Gammaproteobacteria* in both soil and cabbage root to the clubroot disease. *Gammaproteobacteria* has been illustrated to be sensitive to various kinds of soil pollution. For example, in the polycyclic aromatic hydrocarbons (PAHs)-polluted soil, a significantly increased abundance of *Gammaproteobacteria* was observed, which developed a novel bioindicator of the PAHs contamination (Niepceron et al., 2013). *Gammaproteobacteria* was revealed to be enriched in plant roots in acid mine drainage-polluted soil, implying its response to the pollution (Li Y. et al., 2020). Also in animals, *Gammaproteobacteria* had been considered a core taxon in the guts of soil fauna, indicating the environmental concentration of soil pollutants. *Alphaproteobacteria* also belong to the *Proteobacteria* phylum and have been identified as an important endophytic bacterium, which occupied a higher abundance in the HR soil (Ferrando and Fernandez-Scavino, 2013). *Alphaproteobacteria* was reported to mediate the resistance of Poaceae to trace elements in the ultramafic soils (Touceda-Gonzalez et al., 2018). *Bacilli* has been suggested to be closely associated with the spreading of plant disease and it has been widely applied as a biocontrol agent, which could control the fungus and bacterial disease (Marrone et al., 2000; Ma et al., 2018). The abundance of *Bacteroidia* was also found to be correlated with various soilborne diseases. For instance, the increasing abundance of *Bacteroidia* was observed in the dwarf bunt-susceptible varieties of wheat (Xu et al., 2021). *Oxyphotobacteria* was found to deplete in the continuous sugarcane planting yield, which was with acidification and decreased organic matter, C/N, and other nutrient elements (Pang et al., 2021). The different proportions of these bacterial classes between the LR and HR soil–root system also suggested their resistance to the clubroot disease, which makes it possible to apply them in the control of clubroot disease.

### Response of Alpha-Diversity to Clubroot Disease in the Infected Soil and Cabbage Roots

Alpha-diversity could represent microbial communities comprehensively with a series of indexes. More specifically, the richness, diversity, coverage, and evenness of the infected soil and cabbage roots could be indicated through the alpha-diversity index (Schloss et al., 2011).

This study found decreasing bacterial richness in the HR soil–root system, reducing bacterial evolutionary diversity, and elevating coverage in the HR cabbage roots. The evenness was insignificant between the LR and HR soil–root systems. The clubroot disease threatens susceptible species and therefore led to the reducing bacterial richness and diversity. Bacterial diversity is a comprehensive indicator associated with species richness and evenness. Higher bacterial diversity improves the resistance to the occurrence and invasion of plant disease. It has been demonstrated that the increasing diversity of probiotics accelerated microbiome function in the rhizosphere and further promoted the suppression of plant disease by enhancing resource competition and interference of pathogens (Hu et al., 2016). Liu et al. compared the bacterial diversity of citrus canker disease-resistant and -susceptible citrus cultivars and found that the bacterial diversity was associated with disease susceptibility and host resistance (Postel and Mekhaldi, 1987). A previous field trial reported that the application of biological control agent significantly improved the bacterial diversity in the rhizosphere of clubroot disease-infected soil, and therefore, reduced the damage induced by clubroot (Hu et al., 2021). Herein, HR soil and cabbage roots showed a relatively lower bacterial alpha-diversity compared with the corresponding LR samples. The bacterial diversity of cabbage roots was found to be lower than that of soil, indicating the higher susceptibility of cabbage roots to the clubroot disease.

### Response of Bacterial Community Structure to Clubroot Disease in the Infected Soil and Cabbage Roots

The clustering analysis suggested a significant difference in the bacterial community structure between the infected soil and cabbage roots. UniFrac (weighted and unweighted UniFrac), Bray–Curtis, and Jaccard distance are the most commonly used algorithms to indicate the microbial structure. Different algorithms focused on several aspects of the bacterial community structure. Specifically, the unweight UniFrac distance is mainly based on the phylogenetic relatedness between different species, while the weighted UniFrac adds the consideration of species richness (Lozupone and Knight, 2005; Lozupone et al., 2007). The Jaccard distance mainly focused on the proportion of different species, and the Bray–Curtis distance pays more attention to the species richness (Bray and Curtis, 1957).

All algorithms showed no obvious separation of the HR soil from the LR soil, and even the HR was comprised in the LR soil, suggesting similar microbial structures in the LR and HR soil. In other words, the clubroot disease did not change the structure of the bacterial community in the soil. A previous study found a significant difference in the bacterial community structure between the healthy soil and the clubroot disease-infected rhizosphere soil and demonstrated that soil AP and exchangeable calcium were the major affecting factors of the changes (Wu et al., 2020). The differential results of this study might result because (1) both the LR and HR groups were clubroot disease-infected soil with different severity, and therefore, it might be different from the comparison with the healthy soil and (2) no significant difference was observed in the AP and exchangeable calcium between the LR and HR soil, and the bacterial community structure was less affected by the soil physicochemical properties. For the microbial structure of the cabbage roots, both the UniFrac and Jaccard distance algorithms showed a clear separation between the LR cabbage roots and HR cabbage roots, indicating that species richness and phylogenetic relatedness are the major reasons responsible for the different structures between the LR and HR roots.

### Differential Bacteria and Their Relationship With Soil Properties

The abundance of bacteria in the LR and HR soil–root system is related to their resistance to the clubroot disease. Hence, the identification of differential bacteria could filter the resistant bacteria and provide candidates for the biocontrol of clubroot. For instance, Zhou et al. isolated three dominant strains from the clubroot disease-infected rhizosphere soil and used them as the biocontrol strains for the Chinese cabbage clubroot disease. Those two strains showed dramatic biocontrol and promotion effects, which suppressed the incidence and severity of the clubroot disease and significantly improved the growth and resistance of Chinese cabbage (Zhou et al., 2020).

In the clubroot disease-infected soil, *Bacillus, Pseudomonas*, and *B. asahii* were leaked out as the dominant genus and species, respectively. *Bacillus* is a huge family of various species with diverse metabolic characteristics, where a variety of antagonistic substances were produced. Except for the antibacterial and antifungal effects of *Bacillus* on the plant pathogens, some strains of *Bacillus* were reported to promote the growth of plants (Fira et al., 2018). *B. asahii* is a common specie of *Bacillus*, which previously stood out in the alkaline soil applied with organic fertilizer (Feng et al., 2017). The abundance of *B. asahii* in the HR soil was found to be positively associated with soil pH in this study, which might result from its dominant position in the alkaline soil. Although few data were available for the biocontrol effect of *B. asahii*, its significant abundance in the HR soil also implies its great potential of indicating the occurrence and severity of clubroot disease. Like *Bacillus, Pseudomonas* also plays a crucial role in the biocontrol of plant disease and it was also suggested to improve plant health and yield production due to its beneficial metabolites (Raio and Puopolo, 2021). Besides in the HR soil, the differential abundance of *Pseudomonas* was also observed in the HR cabbage roots, but no significant dominant bacterial species were found in this study. *N. caerulescens* was identified as the major differential bacteria in the cabbage roots belonging to the *Nocardioides* genus, which has been revealed to possess resistance to various plant diseases and has been considered as the candidate biocontrol reagent and biofertilization (Battini et al., 2016; Wang et al., 2021). In a prior yield experiment, soil pH was identified as a critical factor that markedly influenced the biocontrol effect of *Heteroconium chaetospira* on clubroot disease together with moisture content and pathogen density (Narisawa et al., 2005). Herein, the abundance of *N. caerulescens* in the HR cabbage roots was also negatively correlated with soil pH. Further establishment of *N. caerulescens*-based bioengineering bacteria should pay more attention to pH conditions.

Although limited evidence has reported the biocontrol effect of the obtained differential bacteria, and there was no significant evidence indicated the function of *B. asahii* and *N. caerulescens* in the occurrence of clubroot disease, their resistance to the severe clubroot disease is undoubted. Deeply, if their genetic mechanism underlying the resistance to the clubroot disease could be disclosed, it will provide more reference for the establishment of correlated bioengineering bacteria. For example, Yu et al. recombineering *Pseudomonas protegens* CHA0, a biocontrol bacterium with a great inhibitory effect on plant disease, with the nitrogen-fixing gene (*nif* ), obtained the mutant strain with bactericidal activity and biological nitrogen fixation (Yu et al., 2019). Therefore, bioengineering bacteria can be established by recombineering functional genes into the dominant bacteria to improve their functions in the clubroot disease-infected soil–root system. Moreover, the differential abundance of *B. asahii* and *N. caerulescens* in the HR soil–root system also implied their potential in serving as indicators of the severe clubroot disease.

Interestingly, among the identified differential bacteria, *B. asahii* was observed in both clubroot disease-infected soil and roots. Hence, it was speculated that *B. asahii* might have spread from the soil to the roots.

### Outlook

Besides the vital role of bacteria in the clubroot disease development in the soil–root system, fungi also play an important role in the microorganism community. Previously, numerous studies have focused on the function of fungi in the spreading of plant disease, especially the diffusion of soilborne disease. The changes in fungi community structure in crucifer clubroot disease were also observed in a previous study (Wu et al., 2020). A huge number of pathogenic fungi that caused plant disease and enlarged disease infection have been disclosed (Busby et al., 2016; Doehlemann et al., 2017). Therefore, the fungi composition of the clubroot disease-infected soil–root system should attract special attention in future studies, which could also provide a novel insight into the biocontrol of clubroot disease.

In contrast, revealing the mechanism of clubroot disease-resistant microorganisms could help understand how bacteria defend against the disease and give more afflatus to improve the tolerance and defense of plants to the clubroot. With the development of bioinformatics, the function enrichment analyses of corresponding genes or species have become a general method, which could leak out the genetic diversity of microorganisms and declaim the potential defense mechanism of resistant bacteria. In the obtained results, three differential bacteria were dug out in the HR clubroot soil–root system. Further investigations on their function could help the development of corresponding bioengineered microorganisms and dramatically benefit the biocontrol of clubroot disease. Moreover, the clubroot disease is mainly induced by the infection of *P. brassicae*. However, no significant difference was observed in the abundance of *P. brassicae* between the LR and HR soil–root systems. The possible reason might be that there was an interaction between *P. brassicae* and the bacterial community, which affected the abundance of *P. brassicae* and the structure of the microbial community in the clubroot disease-infected soil. Therefore, future studies could focus on the effect of *P. brassicae* on soil microbial community and physicochemical properties in order to disclose the pathogenesis of clubroot disease in mechanism and to guide its agricultural control.

## Conclusion

The microbial community composition and structure of infected soil and cabbage roots were closely associated with the severity of the clubroot disease. The lower bacterial diversity and high abundance of *B. asahii* and *N. caerulescens* in the soil–root system were indicators of the high risk of the clubroot disease. The resistance of *B. asahii* and *N. caerulescens* to the clubroot disease suggested their great potential in the biocontrol of the clubroot disease.

## Data Availability Statement

The datasets presented in this study can be found in online repositories. The names of the repository/repositories and accession number(s) can be found in the article/Supplementary Material. The data presented in the study are deposited in the NCBI repository, accession number PRJNA 844280.

## Author Contributions

HN and YZhe: conceptualization. HN: methodology, software, data curation, and visualization. HN, RZ and JS: validation. HN and YL: formal analysis. HN, JL, RJ, XS, YZhe, LT, LL, YD, and TL: investigation. YZha and QT: resources and funding acquisition. HN and YD: writing—original draft preparation. TL, YZha, and QT: writing—review and editing and supervision. All authors have read and agreed to the published version of the manuscript.

## Funding

This research was supported by funding from the National Key R&D Program of China (grant no. 2019YFA0904000), the Recruitment Program of Global Experts (1000 Plan), the Shandong Key Research and Development Program (2019JZZY010724), the Qingdao Science and Technology Benefit People (grant no. 21-1-4-ny-22-nsh), the Natural Science Foundation of Shandong Province (ZR2021QC170), and the Program of Introducing Talents of Discipline to Universities (B16030).

## Conflict of Interest

HN, RZ, JS, YL, JL, RJ, XS, YZ, LT, and LL are employed by Qingdao Hexie Biotechnology Co., Ltd. The remaining authors declare that the research was conducted in the absence of any commercial or financial relationships that could be construed as a potential conflict of interest.

## Publisher's Note

All claims expressed in this article are solely those of the authors and do not necessarily represent those of their affiliated organizations, or those of the publisher, the editors and the reviewers. Any product that may be evaluated in this article, or claim that may be made by its manufacturer, is not guaranteed or endorsed by the publisher.

## Supplementary Material

Supplementary Figure 1 Comparison of the alpha-diversity indexes between the LR and HR soil–root system. ^*^*p* < 0.05, ^**^*p* < 0.01, ^***^*p* < 0.001.

Supplementary Figure 2 Significant differential bacterial genus between the LR and HR soil **(A)** and cabbage roots **(B)**. Different colors represent bacterial abundance.
