# Supplementary figures and images for: Response of Bacterial Community to the Occurrence of Clubroot Disease in Chinese Cabbage
Source: Front Microbiol. 2022 Jul 6;13:922660. doi: 10.3389/fmicb.2022.922660 (PMC9298529; doi:10.3389/fmicb.2022.922660)

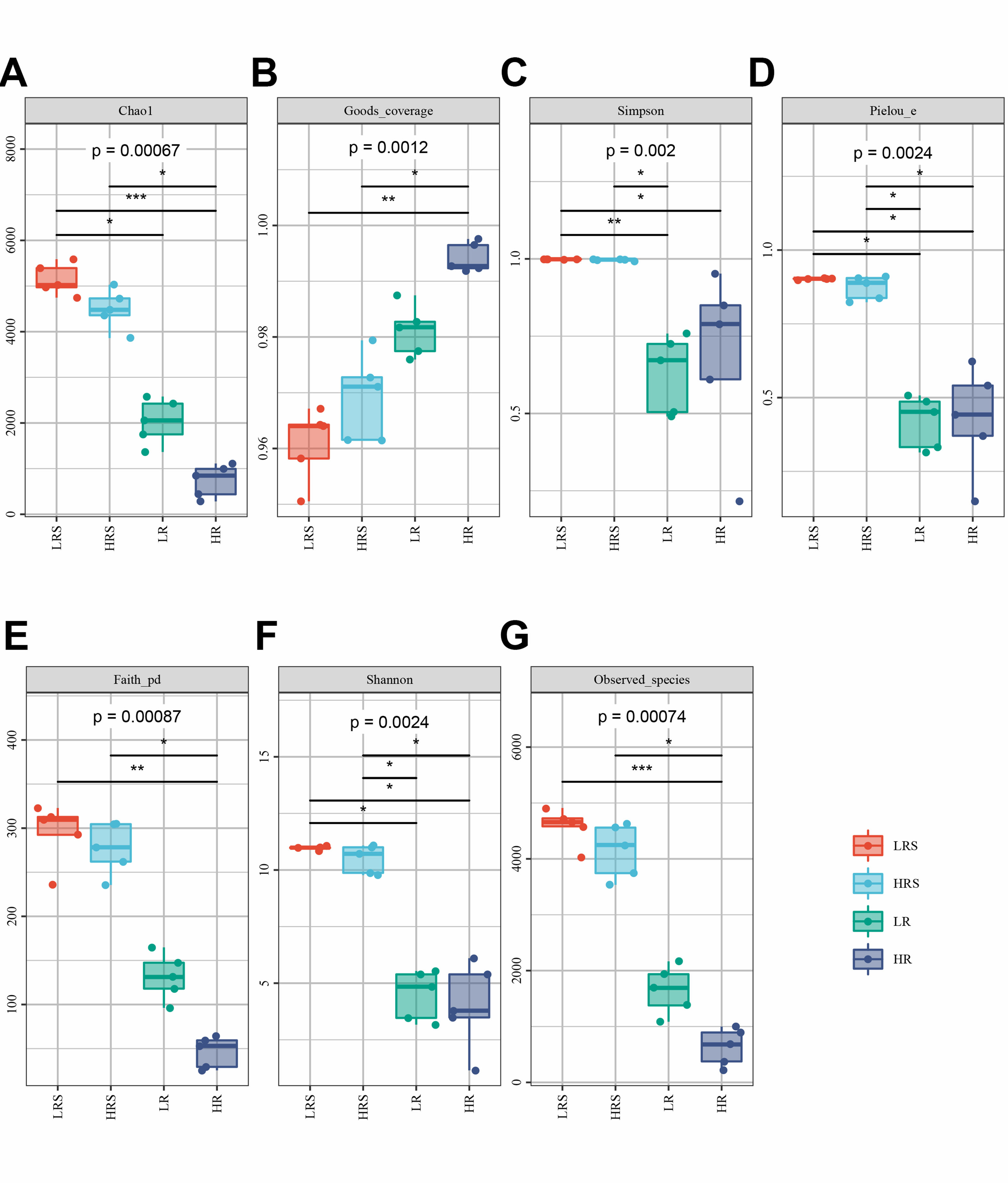

Supplement: Supplementary Figure 1 — Comparison of the alpha-diversity indexes between the LR and HR soil–root system. *p < 0.05, **p < 0.01, ***p < 0.001. [file Image_1.jpg]

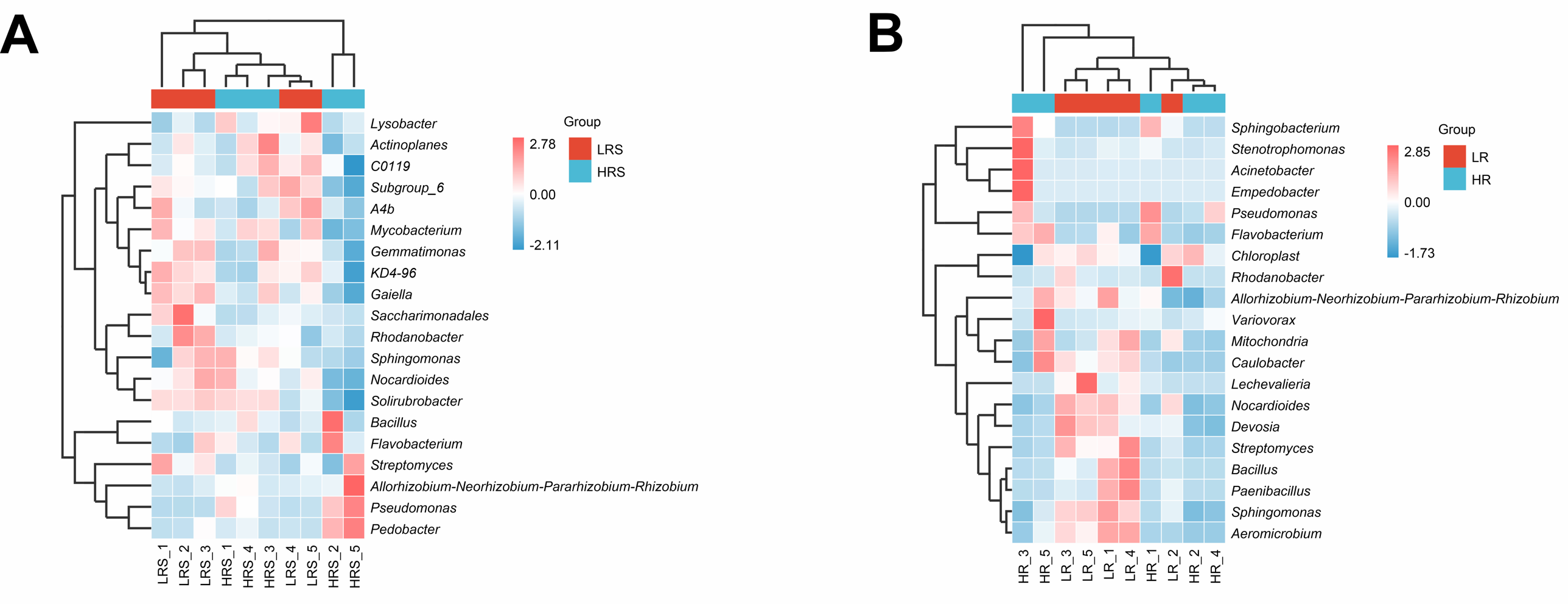

Supplement: Supplementary Figure 2 — Significant differential bacterial genus between the LR and HR soil (A) and cabbage roots (B). Different colors represent bacterial abundance. [file Image_2.jpg]
